# Safety and Outcomes of Amikacin Liposome Inhalation Suspension for *Mycobacterium abscessus* Pulmonary Disease: A NTM-NET study

**DOI:** 10.1016/j.chest.2022.01.015

**Published:** 2022-01-19

**Authors:** Sanne Maria Henriette Zweijpfenning, Raphael Chiron, Sharon Essink, Jodie Schildkraut, Onno W. Akkerman, Stefano Aliberti, Josje Altenburg, Bert Arets, Eva van Braeckel, Bénédicte Delaere, Sophie Gohy, Eric Haarman, Natalie Lorent, Genevieve McKew, Lucy Morgan, Dirk Wagner, Jakko van Ingen, Wouter Hoefsloot

**Affiliations:** a Department of Pulmonary Diseases, Radboud University Medical Centre, Radboud University Medical Centre Dekkerswald, Nijmegen, The Netherlands; b University Medical Centre Montpellier, Montpellier, France; c Department of Medical Microbiology, Radboudumc Centre for Infectious Diseases, Radboud University Medical Centre, Nijmegen, The Netherlands; d Department of Pulmonary Diseases, University Medical Centre Groningen, Groningen, The Netherlands; e TB Center Beatrixoord, University Medical Centre Groningen, Groningen, The Netherlands; f Department of Biomedical Sciences, Humanitas University, Milan, Italy; g Respiratory Unit, IRCCS Humanitas Research Hospital, Milan, Italy; h Department of Pulmonary Diseases, Amsterdam University Medical Centre, Amsterdam, The Netherlands; i Emma Children’s Hospital, Amsterdam University Medical Center, Location VUmc, Amsterdam, The Netherlands; j Department of Respiratory Medicine, Ghent University Hospital, Ghent, Belgium; k Department of Internal Medicine and Paediatrics, Ghent University, Ghent, Belgium; l Department of Infectious Diseases, University Hospital UCL Namur, Yvoir, Belgium; m Department of Pulmonary Diseases and CF Reference Center, Cliniques Universitaires Saint-Luc, Université Catholique de Louvain, Brussels, Belgium; n Department of Respiratory Diseases, University Hospitals Leuven, Leuven, Belgium; o Department of Microbiology and Infectious Diseases, Concord Repatriation and General Hospital, NSW Health Pathology, Sydney, Australia; p Department of Respiratory Medicine, Concord Clinical School, University of Sydney, Sydney, Australia; q Department of Internal Medicine II, Division of Infectious Diseases, Freiburg University Medical Centre, Freiburg, Germany

To the Editor:

*Mycobacterium abscessus* is an opportunistic pathogen notorious for its antibiotic resistance and poor treatment outcomes.^1^ Treatment regimens for *M abscessus* pulmonary disease consist of an intensive phase of 2 to 3 months of IV antibiotics (including amikacin, imipenem or cefoxitin, and tigecycline) combined with oral drugs (including clofazimine, linezolid, azithromycin). The continuation phase consists of two or three oral antibiotics, preferably with proven in vitro activity, and inhaled amikacin.^1^^,^^2^

Amikacin liposome inhalation suspension (ALIS) allows for better biofilm and macrophage penetration^3^ and is likely more effective than inhalation of the IV solution of the drug. We aimed to assess the safety and outcomes of compassionate use of ALIS in *M abscessus* pulmonary disease.

## Methods

Through the NTM-net (an international network to promote clinically oriented research in the field of nontuberculous mycobacterial diseases around the world [www.ntm-net.org]), we recruited physicians who were experienced in the use of ALIS in *M abscessus* pulmonary disease. Patients were considered eligible if they fulfilled American Thoracic Society/European Respiratory Society/Infectious Diseases Society of America/European Society of Clinical Microbiology and Infectious Diseases diagnostic criteria for NTM lung disease^1^ and had received ALIS for at least 2 weeks.

Pseudonymised information of 41 patients was gathered via an online case report form.

The study was approved by the respective Ethical Committees, and patient informed consent was obtained when required.

Treatment outcomes were defined according to the NTM-net statement^4^ for which the outcomes cure, microbiologic cure, and clinical cure were combined into “good outcome.” Drug susceptibility was defined according to international guidelines.^1^

## Results

Forty-one patients from five different countries were included in the study. Their baseline characteristics are presented in Table 1. Cystic fibrosis (CF) and non-CF bronchiectasis were the most common predisposing conditions (51.2%; 31.7%); nodular-bronchiectatic disease was the most prevalent disease manifestation (73.1%). The majority of the patients were infected with *M abscessus* subsp *abscessus*. Six of the isolates were amikacin resistant (14.6%), and 56.1% were macrolide resistant. Most patients were initiated on ALIS not only because of toxicity of IV amikacin (n = 10; 24.4%) but also to strengthen the oral regimen in the continuation phase (n = 6; 14.6%) for treatment of refractory *M abscessus* disease (n = 8; 19.5%) and other/unknown reasons (n = 17; 41.5%). Toxicity of IV amikacin consisted of ototoxicity (n = 8), renal toxicity unspecified toxicity (both n = 1). One patient with ototoxicity with IV amikacin also experienced ototoxicity with ALIS.

**Table 1:** Baseline Characteristics and Treatment Outcomes

Characteristic	Overall (N = 41)	Patients With Detailed Treatment History (n = 26)
Male	23 (56.1)	12 (46.2)
Age, mean ± SD, y	40.3 ± 22.1	41.6 ± 21.2
Country		
Netherlands	12 (29.3)	10 (38.5)
Belgium	8 (19.5)	8 (30.8)
France	18 (43.9)	5 (19.2)
Italy	1 (2.4)	1 (3.8)
Australia	2 (4.9)	2 (7.7)
Smoking		
Never Smoker	29 (70.7)	16 (61.5)
Smoker	2 (4.9)	2 (7.7)
History of smoking	10 (24.4)	8 30.8)
Comorbidity		
Cystic fibrosis	21 (51.2)	11 (42.3)
COPD	5 (12.2)	5 (19.2)
Asthma	5 (12.2)	1 (3.8)
Non-cystic fibrosis bronchiectasis	13 (31.7)	9 (34.6)
Gastroesophageal reflux	3 (7.3)	2 (7.7)
Radiologic presentation		
Nodular-bronchiectatic disease	30 (73.2)	17 (65.4)
Fibrocavitary disease	8 (19.2)	7 (26.9)
Unknown	3 (7.3)	2 (7.7)
*M abscessus* subspecies		
*abscessus*	25 (61.0)	14 (53.8)
*bolletii*	3 (7.3)	2(7.7)
*massiliense*^a^	2 (4.9)	2 (7.7)
unknown	11 (26.9)	8 (30.8)
Susceptibility testing		
Macrolides		
Susceptible	10 (24.4)	10 (38.5)
Resistant (including inducible resistance)	23 (56.1)	15 (57.7)
Unknown	7 (17.1)	1 (3.8)
Amikacin		
Susceptible	15 (36.6)	14 (53.8)
Intermediate	5 (12.2)	4 (15.4)
Resistant	6 (14.6)	4 (15.4)
Unknown	15 (36.6)	4 (15.4)
Copathogens		
*Aspergillus* spp	16 (39.0)	11 (42.3)
*Pseudomonas aeruginosa*	15 (36.6)	6 (23.1)
*Staphylococcus aureus*	13 (31.7)	8 (30.8)
*Achromobacter xylosoxidans*	4 (9.8)	3 (11.5)
*Stenotrophomonas maltophilia*	3 (7.3)	3 (11.5)

	All Patients (N = 41)	*M abscessus* subsp. *abscessus* (n = 25)
Treatment ALIS ongoing	18 (43.9)	9 (36.0)
Previous treatment with IV amikacin	37 (90.2)	21 (84.0)
Mean duration of NTM treatment before start of ALIS, mean ± SD, mo	12.7 ± 19.6	15.8 ± 22.6
Mean duration of treatment with ALIS, mean ± SD, mo	12.4 ± 11.4	13.6 ± 11.6
Culture conversion	18 (43.9)	11 (44.0)
Good outcome^a^	25 (61.0)	15 (60.0)
Microbiologic cure	8 (19.5)	3 (12.0)
Clinical cure	7 (17.1)	4 (16.0)
Cure	10 (24.4)	8 (32.0)
Treatment failure	13 (31.7)	9 (36.0)
Death	2 (4.9)	1 (4.0)
Unknown	1	0

	Cystic Fibrosis (n = 21)	Non-Cystic Fibrosis (n = 20)
Culture conversion	9 (42.9)	9 (45.0)
Good outcome^b^	10 (47.6)	15 (75.0)
Microbiologic cure	3 (14.3)	5 (25.0)
Clinical cure	1 (4.8)	6 (30.0)
Cure	6 (28.6)	4 (20.0)
Treatment failure	10 (47.6)	3 (15.0)
Death	1 (4.8)	1 (5.0)
Unknown	0	1 (5.0)

	Nodular Bronchiectatic (n = 30)^b^	Fibrocavitary (n = 8)^c^
Culture conversion	15 (50.0)	3 (37.5)
Good outcome^b^	19 (63.3)	4 (50.0)
Microbiologic cure	5 (16.7)	3 (37.5)
Clinical cure	4 (13.3)	1 (12.5)
Cure	10 (33.3)	0
Treatment failure	10 (33.3)	2 (25.0)
Death	1 (3.3)	1 (12.5)
Unknown	0	1 (12.5)

Data are presented as No. (%), unless otherwise noted. Treatment outcomes were divided into all patients and *M abscessus* subspecies *abscessus* caused by inducible macrolide resistance and the possibility of poorer outcomes in this group.

a Both strains of *M abscessus* subsp *massiliense* were susceptible to clarithromycin.

b Good outcome = microbiologic cure + clinical cure + cure.

c Three patients had unknown radiologic presentation, of which two patients had clinical cure, and one patient had treatment failure. ALIS = amikacin liposome inhalation suspension; NTM = nontuberculous mycobacteria.

**Figure 1:**
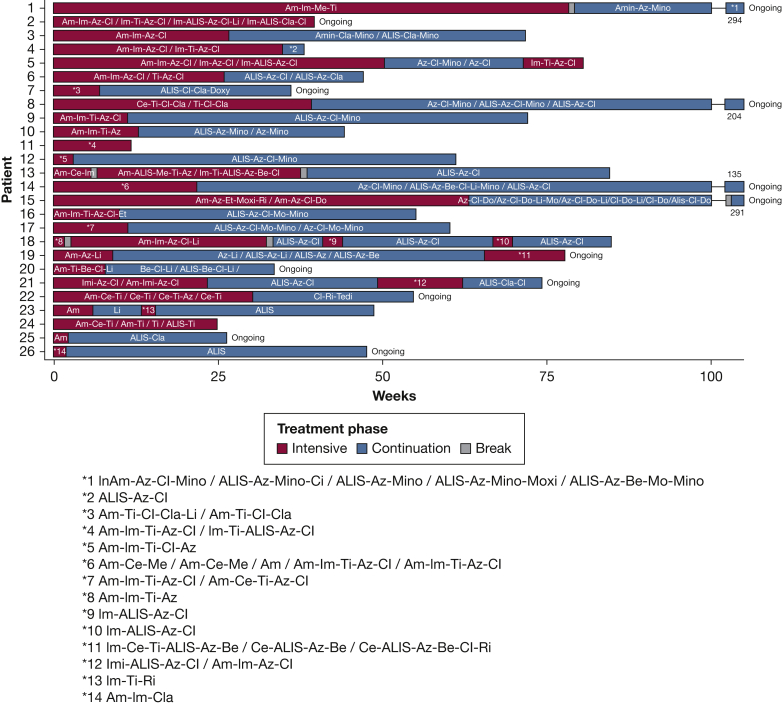
For 26 patients, detailed treatment information was available. Treatment during the intensive phase and the continuation phase are specified and include antibiotic regimens. Breaks in the regimen are of unspecified length. IV antibiotics consisted of amikacin, cefoxitin, imipenem, meropenem, and tigecycline. Oral antibiotics consisted of azithromycin, bedaquiline, clarithromycin, clofazimine, doxycycline, linezolid, minocycline, moxifloxacin, and rifamycin. Inhaled antibiotics consisted of amikacin inhalation (not ALIS), liposomal amikacin inhalation. Treatment either ended or was still ongoing. ALIS = liposomal amikacin inhalation; Am = amikacin; Amin = amikacin inhalation (not ALIS); Az = azithromycin; Be = bedaquiline; Ce = cefoxitin; Cl = clofazimine; Cla = clarithromycin; Do = doxycycline; Im = imipenem; Li = linezolid; Me = meropenem; Mino = minocycline; Mo = moxifloxacin; Ri = rifamycin; Ti = tigecycline.

Detailed information about antibiotic treatment regimens was available from 26 patients (Fig 1). No differences in baseline characteristics were seen between this subgroup and the total group of patients. In Table 1, these patients were included separately to specify outcomes for this group.

Culture conversion was attained in 43.9% of patients. Twenty-five patients (61.0%) had a good outcome (Table 1). Treatment failure was observed in 13 patients (31.7%); death occurred in two patients (4.9%), and the outcome was unknown in one patient. Two of the six patients with amikacin-resistant isolates had a good outcome (33.3%). No differences in terms of good outcome were seen between macrolide-susceptible and resistant strains.

Patients with CF showed a trend towards poorer treatment outcomes than did patients without CF (Table 1). Among patients with CF, 47.6% had a good outcome vs 75.0% in patients without CF (*P* = .069). No differences in cure rates were seen between fibrocavitary and nodular bronchiectatic disease (Table 1).

Adverse events related to ALIS administration were reported in 65.9% of patients; the most frequently reported adverse events were cough (n = 18; 43.9%), dyspnea (n = 9; 22.0%), and ototoxicity (n = 9; 22.0%). Six patients (14.6%) stopped ALIS treatment because of adverse events.

## Discussion

We present a relatively large cohort of patients with *M abscessus* pulmonary disease who were treated with ALIS in addition to their multidrug regimen on compassionate use basis. Although biased by differences in timing and indication for ALIS use, a good outcome was observed in 61% of the patients for whom sufficient data were available.

The observed toxicity (cough, 44%; dyspnea, 22%; ototoxicity, 22%; and discontinuation rate, 15%) in our study is comparable with a phase III randomized controlled trial of the use of ALIS in refractory *M avium* complex pulmonary disease (with cough, 37%; dyspnea, 22%; hearing loss, 4.5%; and tinnitus 8%, respectively).^5^ The 15% discontinuation rate suggests that the adverse events were manageable without requiring treatment cessation.

One randomized, placebo, controlled phase II study assessed the efficacy of ALIS in 32 patients with refractory *M abscessus* with and without CF.^6^ Four of 32 patients achieved culture conversion (three patients received ALIS; one patient received placebo). A case series in patients with CF from France reported clinical improvement and *M abscessus* culture conversion in three of five patients.^7^ Compared with non-ALIS-containing regimens, our observed culture conversion rate of 43.9% seems similar compared with rates that ranged from 34% to 51% in recently published case series and meta-analyses.^8^^,^^9^ Although amikacin levels after ALIS inhalation in fibrocavitary lesions have been shown to be low and possibly subtherapeutic,^10^ we found no significant difference in outcome between the fibrocavitary and nodular bronchiectatic phenotype, which might be biased because only a very small proportion of the patients had fibrocavitary disease. It is possible that, when more patients are included, this difference does become clear. Furthermore, underlying disease such as CF may play a larger role in outcome. Most of the patients with fibrocavitary disease did not have CF (62.5%). Another contributing factor is the effect of the accompanying drug regimens; possibly ALIS plays only a small role in outcome. In patients with CF within the current cohort, 42.9% reached culture conversion, which corresponds with previous data from systematic reviews: 45%.^8^^,^^9^ We recorded no significant difference in culture conversion rates between patients infected with amikacin-resistant or amikacin-susceptible strains, although numbers were too small to allow for meaningful analyses.

Data from this study should be interpreted with caution because of the small yet heterogeneous cohort. Furthermore, ALIS was incorporated rather late in the treatment course because of limited access; in very different regimens, that may have been started with different goals (eradication, suppression, or other). Hence, the efficacy of ALIS in the treatment of *M abscessus* pulmonary disease cannot be estimated readily from the data presented in this report.

In conclusion, ALIS showed manageable respiratory adverse events; outcomes were in line with outcomes of guideline-based treatments for *M abscessus* pulmonary disease. Given the variability in treatment regimens, the additive effect of ALIS is difficult to determine. Based on these results, a clinical trial with an ALIS-containing regimen for *M abscessus* pulmonary disease seems warranted; considering the experiences collected within this cohort and existing guidelines,^1^ its primary role could be in the continuation phase of treatment, as a companion to at least two active oral antibiotics.
